# Reconceptualizing the sense of agency: expanding Decision-level Agency as mental action in the era of generative AI

**DOI:** 10.3389/fpsyg.2026.1768505

**Published:** 2026-02-25

**Authors:** Gaiqing Kong

**Affiliations:** Department of Psychology, School of Sports Medicine, Wuhan Sports University, Wuhan, Hubei, China

**Keywords:** reconceptualizing the sense of agency, Decision-level Agency, Action-level Agency, Outcome-level Agency, autonomy, generative AI, AI-mediated environments, volition

## Abstract

The sense of agency (SoA) - the experience of controlling one’s actions and, through them, events in the external world - is a cornerstone of cognitive science, psychology, and philosophy, underpinning autonomy and responsibility. Yet research on SoA has overwhelmingly focused on Outcome-level Agency (control over external effects) and, to a lesser extent, Action-level Agency (control over bodily movements). A third, upstream dimension - Decision-level Agency, defined as the experience of originating and committing to one’s own decisions or intentions even in the absence of overt action - has remained comparatively neglected and rarely operationalized as a distinct target of measurement. Drawing on philosophical analysis and converging neuroscientific evidence, this paper argues that deciding and intending constitute mental actions in their own right, as the brain actively selects, commits to, revises, or withholds intentions. I propose a three-level framework - Decision, Action, and Outcome - that explicitly incorporates Decision-level Agency as a distinct yet hierarchically integrated component of SoA. This reconceptualization is not only theoretically informative but ethically urgent in the era of generative artificial intelligence, where external systems can increasingly shape human autonomy upstream at the level of decision formation rather than action execution. By outlining testable predictions and experimental paradigms, this work establishes Decision-level Agency as an empirically tractable dimension of human volition and provides a framework for understanding and safeguarding autonomy in AI-mediated environments.

## 1 Introduction

The Sense of agency (SoA) is fundamental to human subjectivity, currently defined as the experience of controlling one’s actions and, through them, events in the external world (Gallagher, 2000; Haggard, 2017; Haggard and Chambon, 2012). For instance, when you flip a light switch and see the room brighten, or press a piano key and hear a corresponding tone, you not only execute these movements, but also feel that you caused their outcomes - the room brightens or the note sounds because of you. SoA is critical for distinguishing self-generated actions from external events, thereby supporting autonomy, skill acquisition, and the coherent experience of self as an agentive being (Bayne, 2011). It also underpins learning, decision-making, and social interactions, providing a foundation for navigating and shaping one’s environment.

### 1.1 The dominance of motor-centric agency: outcome and action levels

Traditional SoA research has largely been anchored in the study of overt, measurable motor acts and their consequences (Haggard, 2017; Moore and Obhi, 2012). As a result, current theoretical frameworks and empirical measures of SoA systematically privilege two levels of control, even though broader theoretical accounts of agency can, in principle, extend beyond bodily movement.

First, Outcome-level Agency refers to the sense of causing external effects - turning on a light, producing a sound, shifting an object. This level has been the most intensively studied within cognitive science. To understand how this aspect of SoA arises, the comparator model provides a useful theoretical framework, initially developed to account for motor learning and control (Blakemore et al., 1998, 2001; Kawato, 1999; Wolpert et al., 1995). These models propose that SoA emerges when internally generated predictions regarding the sensory consequences of an action align with the actual sensory outcomes (Frith et al., 2000; Haggard, 2017; Synofzik et al., 2008). This alignment between the predicted and actual feedback, such as when turning a knob produces the expected click, fosters the subjective feeling of control over actions and their effects. Conversely, SoA diminishes when actions yield unexpected feedback. However, it is important to note that recent studies indicate SoA is a complex phenomenon involving multiple processes beyond the simple comparator mechanism, including action selection, intention, effort, emotion, and social interaction (Caspar et al., 2016; Chambon et al., 2013; Moore, 2016; Sidarus and Haggard, 2016). Furthermore, cue integration approaches argue that the brain weights multiple sources of evidence - including motor signals, sensory cues, and contextual information - to infer authorship (Moore and Fletcher, 2012). Overall, research confirms that SoA operates at both sensorimotor and cognitive levels, necessitating varied measurement approaches (Synofzik et al., 2008).

The second dimension, Action-level Agency, concerns the experience of initiating or controlling bodily movement, irrespective of external consequences (Chambon et al., 2014; Pacherie, 2008; Wen, 2019), for example, the fluency of a hand gesture. This action-level dimension closely corresponds to what has been described as body/self agency, namely the experience of being the agent of one’s own bodily action, as distinct from agency over external events or outcomes (Farrer and Frith, 2002; Sperduti et al., 2011; Wen, 2019). At this level, agency is typically grounded in processes related to motor preparation, initiation, and online control of bodily movement, drawing on predictive motor signals (e.g., efference copy–based expectations), bodily sensory feedback (e.g., proprioception), and the monitoring of movement fluency during execution. In this sense, Action-level Agency reflects control and authorship over “how the movement unfolds,” whereas Outcome-level Agency concerns the causal attribution of external consequences produced by that movement. Although Action-level Agency has received comparatively less attention than outcome-level control, it remains inherently motoric: action is conceptualized primarily as movement and agency as control over that movement.

Together, these two dominant levels have encouraged a presupposition in much of the empirical literature: that agency is primarily grounded in motor execution and is most readily expressed when actions unfold and generate observable consequences. Importantly, however, agency-related research sometimes extends beyond overt movement, for example, studies on vicarious agency when observing others’ predictable actions (Wegner et al., 2004; Weiss and Schütz-Bosbach, 2012), as well as research on the intention not to act (Kühn et al., 2009). Nonetheless, most standard SoA paradigms remain motor-anchored. This emphasis has shaped not only the design of experimental paradigms but also the conceptual boundaries of the field.

### 1.2 Limitations of current measurement paradigms

Current empirical research on SoA typically employs two measurement approaches: explicit and implicit measures (Dewey and Knoblich, 2014; Kong et al., 2017; Moore et al., 2012; Moore and Obhi, 2012). Explicit measures involve tasks where participants are required to consciously judge their feelings of control over specific sensory events or to explicitly identify whether they authored an action (Kokkinara et al., 2015, 2016; Ma and Hommel, 2015; Tieri et al., 2015). These judgments provide direct access to participants’ self-attributed sense of agency but may also be influenced by strategic or reflective processes. In contrast, implicit measures seek to capture automatic processes underlying SoA without requiring explicit judgments. The intentional binding effect (also more generally referred to as temporal binding) is one of the most commonly used implicit measures of SoA. This effect refers to the phenomenon where individuals perceive the temporal interval between their voluntary action and its consequence as shorter than the interval between two events in which their voluntary actions were not involved (Haggard et al., 2002; Moore and Obhi, 2012). Beyond intentional binding, other implicit measures have also been employed to study different facets of SoA. For example, sensory attenuation refers to the phenomenon where self-generated sensory feedback is perceived as less intense compared to externally generated feedback, reflecting the brain’s ability to predict and modulate the sensory consequences of its own actions (Blakemore et al., 1998; Shergill et al., 2003).

While the existing paradigms, which rely on either explicit judgment or implicit measures, have provided valuable insights into the SoA, they are subject to several key limitations. First, these methods often employ simplistic tasks, such as button presses that produce simple tones or flashes, which, though well-controlled, fail to capture the complexity and dynamism characteristic of real-world volition and action. Second, there is a critical measurement confound inherent in implicit measures. Temporal binding is increasingly recognized as a multifaceted phenomenon. Recent studies have demonstrated that temporal binding may arise from factors such as causality, predictability, somatosensory integration and spatial attention, rather than uniquely reflecting the intentionality and voluntariness of the action itself (Buehner, 2015; Buehner and Humphreys, 2009; Dewey, 2024; Dewey and Knoblich, 2014; Gutzeit et al., 2023; Kirsch et al., 2019; Kong et al., 2024; Suzuki et al., 2019). This complexity compromises its utility as a pure implicit measure of agency. Third, empirical research frequently encounters a disassociation problem, where explicit and implicit measures of SoA yield dissociated and uncorrelated results (Dewey and Knoblich, 2014; Saito et al., 2015; Siebertz and Jansen, 2022), indicating that current methods may capture different, unrelated facets of agency, suggesting the lack of a unified, comprehensive theoretical framework.

### 1.3 The neglect of Decision-level Agency

Most critically, existing measures of the SoA are constrained by strong conceptual tethers: they implicitly bind agency to observable motor output and its external effects. This theoretical commitment systematically excludes forms of agency that arise before action occurs. As a result, the upstream internal processes of intention and decision formation remain largely unaccounted for. In traditional SoA theories, decisions are typically treated as pre-motor inputs to action but not as agentive events in their own right. This bias obscures a crucial locus of autonomy. Philosophical analyses (Davidson, 1963; Mele, 2003; Shepherd, 2014) and neuroscientific evidence (Brass and Haggard, 2008; Ridderinkhof, 2014; Ridderinkhof et al., 2004) instead emphasize that multiple forms of control are exercised prior to motor execution. These upstream processes are where autonomy is initiated, not merely where it is expressed.

**FIGURE 1:**
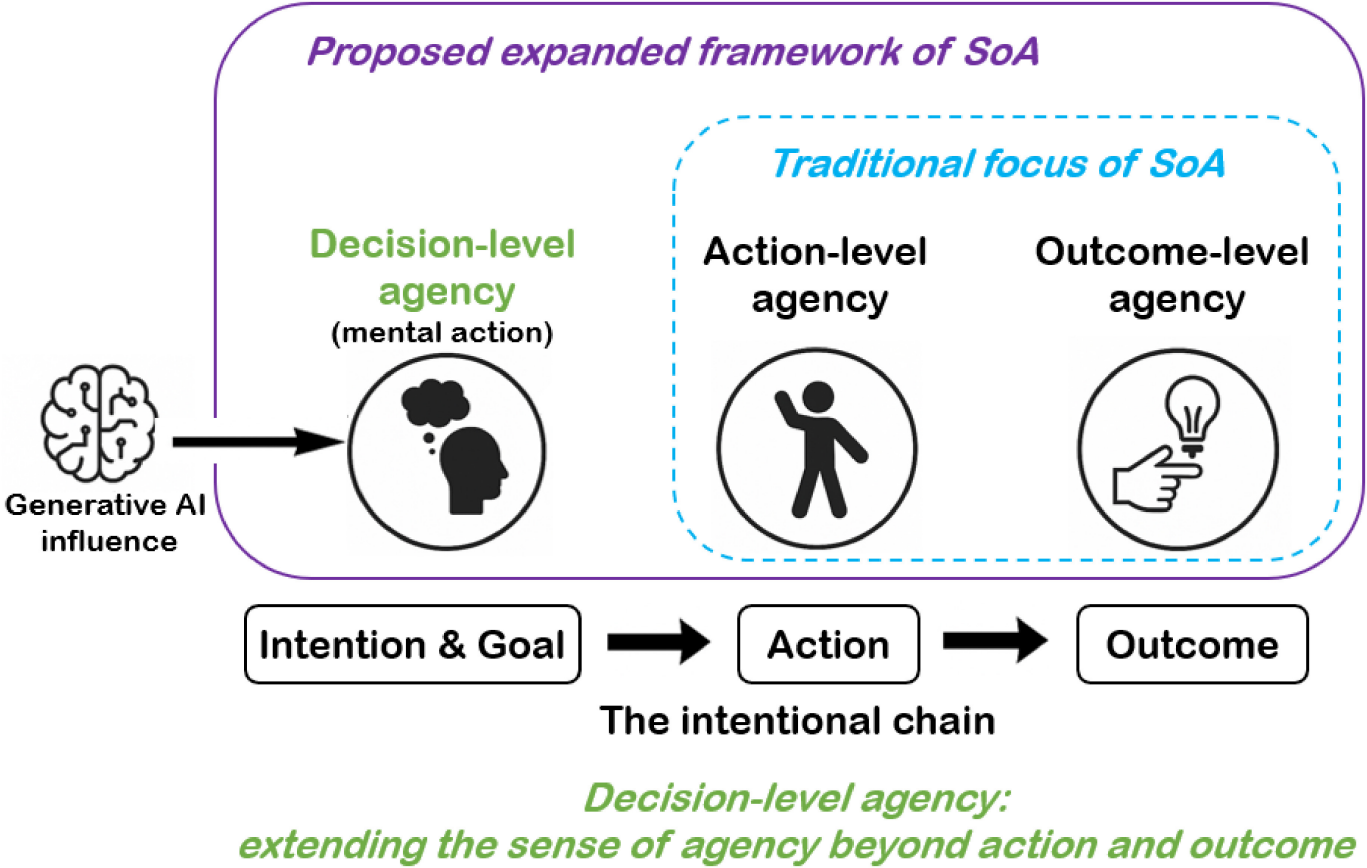
A three-level framework of agency incorporating Decision-level Agency (proposed in the present work). Decision-level Agency corresponds to the experience of originating and committing to an intention and can arise even in the absence of overt movement or external outcomes. Action-level Agency concerns the experience of initiating and controlling bodily movement. Outcome-level Agency concerns the experience of causing external effects.

Despite their centrality to voluntary behavior, SoA research has rarely attempted to isolate or directly measure the subjective experience of originating one’s own decisions or intentions, here that is, Decision-level Agency (Figure 1), although notable exceptions include work showing that coercion can reduce agency-related measures such as intentional binding even when actions are executed normally (Caspar et al., 2016). Many core experiences of self-governance, such as choosing to act, choosing to refrain, resolving internal conflict, or changing one’s mind, are fundamentally non-motoric. Crucially, Decision-level Agency can be present in the absence of any motor preparation or execution, whereas Action-level Agency, by definition, cannot. Overt movement is therefore not a necessary condition for Decision-level Agency: individuals can experience authorship over choosing, committing, revising, or intentionally withholding an intention even when no action is performed and no external outcome occurs. This distinction motivates treating decision-level processes as a separable and foundational dimension of agency rather than as a mere subcomponent of motor control. Yet current models offer no principled account of how agency is experienced in the absence of overt movement. Consequently, SoA research remains unable to capture non-motor forms of self-governance and fails to address how autonomy emerges before any physical action is initiated.

On this basis, the present paper proposes to broaden the empirical and phenomenological scope of SoA research by explicitly foregrounding Decision-level Agency. Rather than suggesting that existing theories deny the relevance of intentions, the proposal is that intention formation and commitment should be treated as an explicit target of agency attribution and measurement, alongside action execution and outcome control. On this view, deciding can thus be regarded as a form of mental action that supports a distinctive experience of authorship (“I decided”), which becomes especially salient when autonomy is shaped upstream (e.g., in AI-mediated environments). Accordingly, the present work demonstrates the critical relevance of Decision-level Agency for understanding and safeguarding human autonomy in the age of generative artificial intelligence.

More broadly, clinical phenomena such as thought insertion and experiences of externally controlled thinking in individuals with schizophrenia illustrate that agency disturbances can arise for purely internal mental events, not only for overt bodily actions. Such cases provide an additional motivation for expanding agency frameworks upstream, toward decision- and thought-level processes, where authorship and commitment may be experienced as compromised even in the absence of overt movement.

## 2 A three-level framework of agency

I propose a formal three-level framework to delineate the domain of SoA and to make explicit the frequently overlooked role of decision-level processes in shaping the SoA experience. Although decision-related internal processes have been discussed theoretically (Gallagher, 2000), they are rarely isolated and operationalized as a distinct level of SoA measurement. Importantly, Decision-level Agency is not introduced as a competing alternative to existing SoA frameworks. Instead, it functions as a conceptual clarification and organizing lens that makes explicit a frequently under-foregrounded locus of agency (intention formation and commitment), thereby enabling more precise empirical dissociations across the decision–action–outcome sequence. Accordingly, conceiving the intentional process as a chain, Decision → Action → Outcome (Figure 1), captures the full trajectory of voluntary behavior, from the origination and stabilization of an intention to its motor implementation and subsequent effects in the external world.

Earlier philosophical analyses (Bayne, 2011; Pacherie, 2008), predictive modeling approaches (Dutta, 2025; Martin and Pacherie, 2019; Ridderinkhof, 2014), and hierarchical action-control accounts (Brass and Haggard, 2008) have discussed multi-stage aspects of action and control. However, these accounts have not explicitly formalized a three-level architecture of agency that assigns distinct phenomenological dimensions to decision formation and intention commitment, action execution, and outcome attribution. The present framework therefore enables a principled differentiation between agency experienced during decision-making as mental action, during movement initiation and control, and during the attribution of external effects. In doing so, it provides a coherent conceptual basis for analyzing how agency is constructed across the full decision-action-outcome sequence (Figure 1).

The three levels are conceptually independent yet hierarchically organized (Table 1). Decision-level Agency, which involves the selection and commitment to a high-level goal or intention, precedes and constrains the subsequent action and outcome levels. In this hierarchy, the output of the decision process functions as the initiating command for action-level processes (motor preparation, initiation, and execution), which in turn generate the external sensory consequences evaluated by the outcome-level system.

**TABLE 1:** Formalization of Decision-level, Action-level, and Outcome-level Agency within the proposed framework.

Dimension	Definition	Nature of control	Examples of subjective experience	Status in current research
Decision-level Agency	The experience of origination and commitment to one’s own decisions, choices, or intentions, independent of overt action.	Mental action/volition	“I chose this option.” “I committed to this goal.” “I deliberately refrained from acting.”	Almost absent
Action-level Agency	The experience of initiating or controlling bodily movements.	Motor initiation/fluency	“I initiated my head movement.” “I controlled how my hand moved.” “The movement felt fluent and under my control”	Moderately studied
Outcome-level Agency	The experience of causing external effects or consequences through one’s action.	Causal efficacy	“I made that tone.” “I made the light turn on.” “I caused the cursor to move on the screen.”	Highly dominant

By formally distinguishing these levels, the framework clarifies both what existing theories of agency successfully account for and what they overlook. It thereby underscores the need for targeted empirical investigation of Decision-level Agency and provides a principled foundation for integrating decision-making mechanisms into contemporary theories of agency.

## 3 Decision-making as mental action

Expanding the sense of agency to include the decision level rests on the recognition that intention formation is not merely a pre-motor event but an active, agent-driven mental operation. Decisions can therefore be understood as mental actions that fundamentally transform an agent’s internal state by selecting, committing to, revising, or withholding an intention.

For clarity, this paper uses the following terms in a consistent way throughout the manuscript. Decision-making refers to the broader process of deliberation and selection among alternatives. Mental action refers to the agentive character of this process when it is intentionally initiated and guided by the individual (e.g., choosing, revising, or withholding an intention). Intention commitment refers to the stabilized outcome of decision formation, namely the transition from multiple candidate intentions to a selected and maintained intention. Finally, policy inference denotes the computational description of this decision-level process within predictive-processing frameworks, whereby candidate policies are evaluated and uncertainty is reduced until a commitment stabilizes. Decision-level Agency is thus understood as the subjective experience associated with this process of intention formation and commitment.

### 3.1 Philosophical and conceptual foundations

Philosophical traditions have long recognized deciding and intending as distinct forms of action that warrant a sense of ownership and authorship. For instance, Davidson (1963) described decisions as intentional acts that commit an agent to a future course of behavior. Searle (1983) similarly argued that forming an intention is an active commitment that transforms an internal state into an action-guiding directive. Mele (2003) and Shepherd (2014) further contended that decisions themselves constitute genuinely agentive operations. Bayne (2011) emphasized the importance of distinguishing between the “experience of deciding” and the “experience of acting.”

On these views, the brain is continuously acting when it selects, revises, or withholds an intention. If agency is inherently tied to action, then the arbitrary exclusion of mental actions (deciding, intending) from the scope of SoA leaves current accounts conceptually incomplete. Recognizing the active nature of decision-making helps resolve this limitation, and extends the domain of agency beyond motor execution to the cognitive sphere where autonomy is often exercised most critically.

### 3.2 Neuroscientific convergence

Converging neuroscientific evidence supports the action-like nature of decision formation by showing that neural processes underlying choice commitment parallel those involved in motor action. Research on the neural precursors of volition demonstrates that activity in prefrontal and parietal cortices tracks the formation, maintenance and updating of intentions (Fried et al., 2011; Soon et al., 2008, 2013). Neural signals traditionally associated with motor preparation - most notably the readiness potential (RP), referring to a slow build-up of negative EEG potential originating from pre-motor cortical areas (Kornhuber and Deecke, 1965; Libet et al., 1983; Shibasaki and Hallett, 2006) - precede before the reported awareness of an intention to move. In Libet et al.’s (1983) paradigm, participants reported the time on a rotating clock when they first became aware of their intention to act (referred to as the will-time or W-time) and RP activity was observed prior to this reported moment. Importantly, the functional interpretation of RP remains debated, and it should not be treated as a direct neural marker of the conscious experience of deciding.

In the present framework, RP-related activity is not be taken to constitute the decision itself, but rather to reflect preparatory dynamics associated with intentional engagement and emerging commitment that may occur before, alongside, or even without overt movement execution. Consistent with this broader interpretation, RP-like components have also been observed during non-motoric and purely cognitive decision processes (Alexander et al., 2016; Schmidt et al., 2016), as well as during intentional inhibition (Schultze-Kraft et al., 2016, 2021), in which a prepared movement is withheld and no action is ultimately executed. Crucially, the presence of RP-like activity in such contexts does not imply that the subjective experience of deciding coincides temporally with these early neural dynamics. Rather, Decision-level Agency is hypothesized to arise when intention formation becomes sufficiently stabilized, i.e., when commitment is reached, which may occur downstream from, or only partially overlap with, preparatory activity. In this sense, Decision-level Agency is neither a purely prospective readout of early neural precursors nor a purely retrospective judgment, but is expected to track the transition from undecided states to a committed intention that is experienced as one’s own decision. These findings therefore suggest that the neural signature of “gearing up to act” can also accompany “gearing up to decide,” supporting the view that decision formation engages action-like neurocognitive dynamics.

Complementing these neural findings, recent behavioral evidence suggests that a measurable sense of agency can arise for purely mental actions in the absence of motor components. Using a belief-based action–effect paradigm, Lopez-Sola et al. (2021) showed that participants reported a comparable explicit sense of agency when they believed that either a motor action or a specific thought triggered an auditory outcome. These results highlight that intentional and inferential cues can instantiate an experience of agency even without overt movement, providing empirical support for the claim that decision-related mental operations can carry an agentive phenomenology in their own right.

More broadly, decision-making engages neural dynamics characteristic of action-selection circuitry, including competitive accumulation in frontoparietal and basal ganglia networks (Cisek and Kalaska, 2010; Forstmann et al., 2008, 2016; Thura and Cisek, 2014). These processes involve predictive coding, evidence accumulation, conflict monitoring, and executive control - mechanisms extensively studied in the decision-making literature and also often implicated in selecting and initiating motor actions. Such overlap suggests that decision formation and motor control may share general computational principles (e.g., prediction, selection, and control), consistent with broader proposals that internal-model mechanisms can generalize beyond the motor domain to cognition (Ito, 2008).

Neuroimaging work and integrative reviews have also identified a distributed network supporting agency in bodily action, including fronto-parietal and midline structures involved in motor prediction, monitoring, and self-attribution (e.g., David et al., 2008; Zito et al., 2020). In particular, meta-analytic evidence highlights a key role for temporo-parietal regions (notably the TPJ) when agency is disrupted, alongside broader contributions from premotor, supplementary motor, prefrontal, parietal, and insular systems implicated in motor control and agency judgments (Zito et al., 2020). Importantly, the present framework does not assume that Decision-level Agency depends on an entirely separate neural system from action-related agency. Rather, it is hypothesized to arise from higher-level control and commitment processes–primarily supported by prefrontal and fronto-parietal networks–that constrain and structure downstream motor preparation and execution. In this sense, decision-level mechanisms are embedded within a broader hierarchy of voluntary control, operating upstream of action-level processes while partially overlapping with the neural circuitry typically implicated in bodily agency.

Taken together, these findings suggest that decision formation engages action-like dynamics that prepare and stabilize commitments, thereby shaping downstream motor implementation. In the present framework, shared computational principles do not imply that decisions and actions occupy the same hierarchical level; rather, decisions operate upstream by constraining what actions are initiated and how they unfold. Based on this integrated evidence, the present framework rests on three core claims: (1) decision-making constitutes a form of mental action and can generate a distinct, measurable sense of agency, including in the absence of overt movement or external outcomes; (2) Decision-level Agency operates upstream and can hierarchically constrain Action- and Outcome-level Agency; and (3) disruptions at the decision level can compromise autonomy even when action execution and outcome control remain intact. Together, these claims provide a structured basis for empirically investigating Decision-level Agency and its role in human volition. Decision-level Agency should not be conflated with purely *post hoc* metacognitive evaluation or confidence judgments. Instead, it refers to the subjective experience of originating and committing to an intention prior to action execution and outcome monitoring.

## 4 Integrating Decision-level Agency with predictive processing

The predictive processing (PP) framework (Clark, 2013; Friston, 2010) conceptualizes the brain as a hierarchical system that continuously generates predictions and minimizes prediction errors across multiple representational levels. This architecture provides a powerful theoretical foundation for integrating Decision-level Agency into contemporary cognitive neuroscience. Within PP, the brain is not a reactive input-output device, but an active inference system that continually constructs and updates internal models to anticipate both external sensory events and internal cognitive states. Prediction errors arise when there is a mismatch between predicted states and the incoming signal, whether sensory, motor or cognitive, and the minimization of these discrepancies is proposed to drive perception, cognition, and action (Pezzulo et al., 2017, 2018; Ridderinkhof, 2014).

**FIGURE 2:**
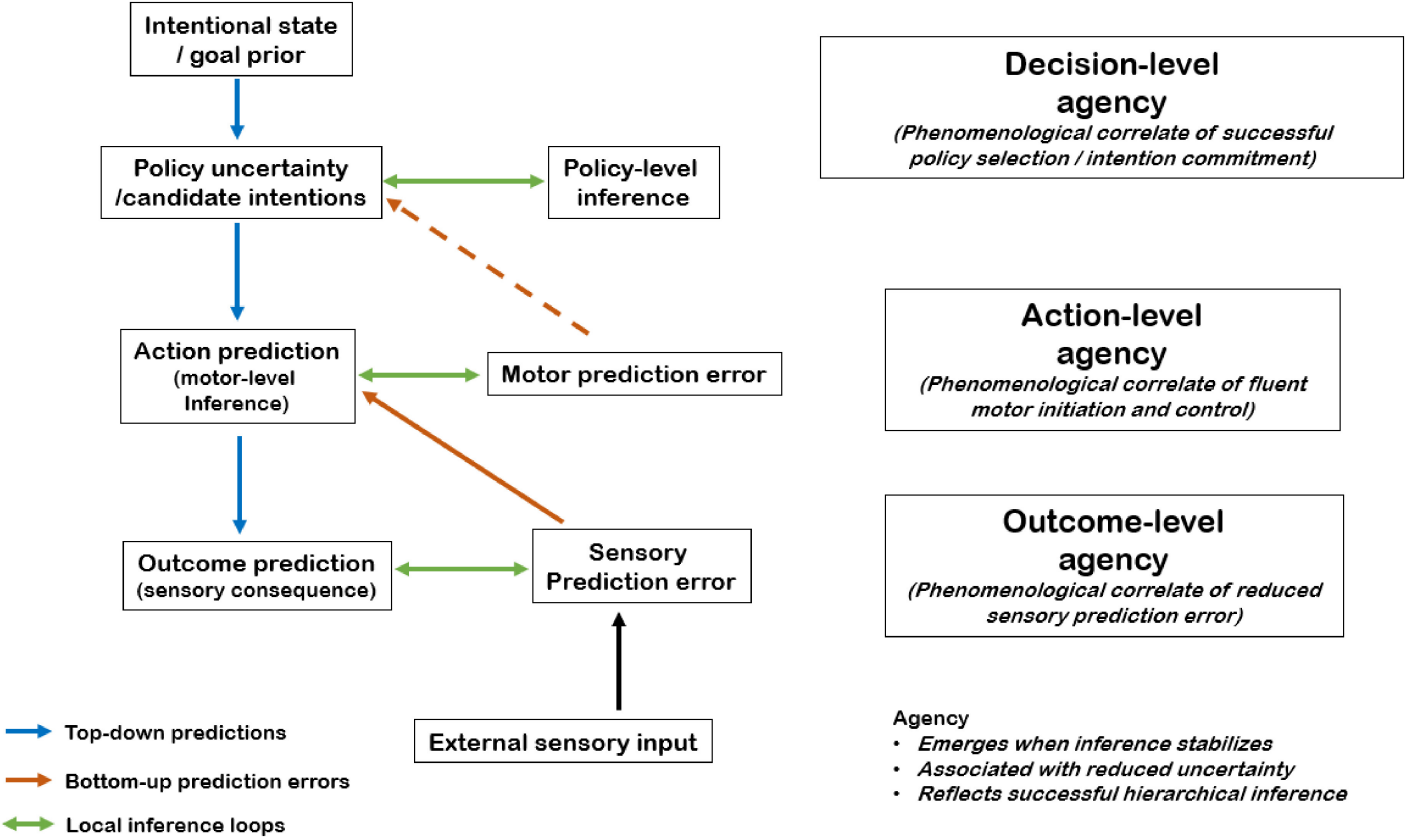
A hierarchical predictive-processing framework for Decision-, Action-, and Outcome-level Agency. The present work proposes a three-level intentional hierarchy in which the sense of agency emerges as a phenomenological correlate of successful inference at distinct stages of voluntary control. At the highest level, intentional states and goal priors generate uncertainty over candidate policies or intentions. Policy-level inference resolves this uncertainty through the evaluation and stabilization of candidate intentions, yielding a committed intention and Decision-level Agency, defined as the subjective experience of originating and committing to one’s own decision, which can occur even when no motor action follows. At the intermediate level, selected policies generate motor-level action predictions, and reduced motor prediction error supports Action-level Agency, corresponding to the experience of fluent motor initiation and control. At the lowest level, predictions about sensory consequences are compared with incoming external sensory input, and reduced sensory prediction error underlies Outcome-level Agency, the experience of causing external effects. Top-down predictions and bottom-up prediction errors interact hierarchically across levels. Dashed bottom-up pathways indicate modulatory constraints whereby motor-level feasibility can influence higher-level policy uncertainty without constituting a primary prediction-error signal. Importantly, agency does not perform error minimization itself but reflects the stabilization of hierarchical inference and reduced uncertainty at each level of the intentional chain.

Although PP has been most extensively applied to sensorimotor control, where it provides a robust account of outcome prediction and underlies classical comparator models, it naturally extends to higher-order cognitive processes and the upper levels of the intentional hierarchy (Chambon et al., 2014; Fleming and Daw, 2017; Frith, 2012). In a PP-informed account of agency, predictions operate at multiple hierarchical stages (Figure 2). At higher levels, the system infers and evaluates candidate policies or intentions, corresponding to prospective courses of action. These decision-level inferences constrain predictions at intermediate motor levels, which specify the actions required to implement a selected intention. In turn, motor-level predictions generate expectations at lower sensorimotor levels concerning the anticipated sensory consequences of action.

This hierarchical architecture allows for a principled formalization of decision-related agency (Figure 2). Just as Outcome-level Agency emerges when predicted and actual sensory consequences align, and Action-level Agency arises when predicted motor states match proprioceptive, haptic or visual feedback, Decision-level Agency can be understood as arising when higher-level policy inference stabilizes. Specifically, Decision-level Agency emerges when uncertainty over candidate intentions is resolved and a particular intention or course of action is successfully selected and committed to, including in cases where no overt action follows. In this case, top-down predictions about one’s own future intentional state align with the internal cognitive dynamics that subsequently unfold, such as the subjective experience of commitment or resolve. Agency is thus grounded not only in accurate predictions of external outcomes or bodily movements but also in the successful internal forecasting and stabilization of one’s own intentions.

To avoid ambiguity, it is important to emphasize that this formulation does not propose an alternative to prediction-error minimization. Rather, within predictive processing, hierarchical inference stabilizes precisely because prediction errors are reduced and uncertainty is resolved across levels. The present proposal is therefore a phenomenological one: the sense of agency is hypothesized to reflect the experiential correlate of successful inference at each level of the hierarchy - namely, the felt reduction of uncertainty and the emergence of stable commitments (at the decision/policy level), fluent control (at the action level), and reliable causal attribution (at the outcome level). In this sense, “stabilization” describes what it is like, from the agent’s perspective, when error minimization has been sufficiently successful to yield a coherent sense of “I decided,” “I acted,” or “I caused.” Predictive processing therefore could offer a unified computational framework that naturally incorporates Decision-level Agency within the broader hierarchy of predictive brain function.

Finally, this perspective may also offer a way to apply the hierarchy to internal mental events. For instance, experiences such as thought insertion or externally controlled thinking may reflect an altered attribution of authorship at the decision/thought level: internally generated contents may be produced normally, yet the system fails to assign them to the self as the source. From a predictive-processing standpoint, this can be conceptualized as atypical high-level priors or precision-weighting over internal generative processes, such that mental events are experienced as “not mine.”

## 5 The urgency: generative AI and the reshaping of autonomy

The need to incorporate Decision-level Agency is not merely theoretical; it is an ethical, societal, and technological imperative driven by the pervasive rise of Generative AI (GenAI). GenAI systems, including large language models (LLMs) akin to GPT-x-like architectures, mark a transformative shift in human-computer interaction by enabling adaptive, context-aware, and increasingly collaborative behavior (Bubeck et al., 2023). Crucially, GenAI increasingly influences human behavior upstream, at the level of intention and decision formation, long before any motor act occurs. This shift represents a profound transformation in human–machine interaction: technologies no longer merely facilitate actions but now shape the choices that give rise to them. In this section, autonomy is understood in two complementary senses: as a subjective experience of authorship over one’s decisions (“I decided”), and as a functional capacity for self-governed intention formation and commitment.

### 5.1 AI intervention at the decision stage

Unlike earlier technologies that primarily mediated, shaped or amplified physical actions, contemporary GenAI systems increasingly intervene upstream in the intentional chain, shaping human decisions as they are formed. Importantly, this intervention differs in kind from more traditional sources of information and guidance (e.g., books, experts, the media, or search engines). Traditional sources typically provide relatively static content that users integrate into their own deliberation, whereas GenAI systems engage in interactive dialogue, adapt to a user’s prior responses, and iteratively refine suggestions in real time. As a result, GenAI can structure deliberation during intention formation by shaping which options are made salient, how trade-offs are framed, and which justificatory narratives appear coherent, thereby influencing how candidate intentions stabilize.

This intervention occurs primarily through framing and suggestion, where AI systems - such as automated recommendation engines, generative drafting tools, and predictive personalization platforms - can bias cognitive priors, narrow the perceived option space, and influence preference formation in real time (Thaler and Sunstein, 2021). By presenting pre-structured choices or suggesting complex decisions that users adopt with minimal reflection and deliberation (Shneiderman, 2020), GenAI challenges human autonomy before any motor output is executed (Hager et al., 2024; Mukherjee and Chang, 2025). For instance, in domains like healthcare, AI diagnostic tools may steer a clinician’s treatment choices, or in policy and governance (Anderson, 2025), algorithmic recommendations may frame available options and decision criteria. In these scenarios, autonomy is not primarily threatened at the level of action execution but at the foundational stage of decision formation, illustrating how GenAI can reshape the intentional process upstream. This motivates the need for SoA frameworks that can capture interference at the decision stage, where AI exerts an increasingly prominent influence.

### 5.2 Ethical and societal implications

If SoA frameworks remain limited to motor and outcome control, they may be insufficient for fully characterizing, measuring, and protecting autonomy in complex AI-mediated environments. Failing to account for Decision-level Agency risks overlooking a major locus of AI influence in the GenAI era. AI-driven “nudging,” automated option framing, or the design of “choice architecture” can subtly diminish genuine agency (Anderson, 2025; Villegas-Galaviz and Martin, 2024), even when the final, overt actions remain voluntary and human-executed. The user may feel they are in control of the action (Who pressed the button?), but the authorship of the intention has already been shaped upstream (Who formed the intention?). Recognizing Decision-level Agency therefore provides a crucial conceptual extension and enables: (1) detection of autonomy-relevant influences that occur before action execution; (2) formal tools for evaluating AI systems in terms of their effects on intention formation and commitment; and (3) normative guidance for designing AI systems that actively preserve, enhance, or restore human Decision-level Agency, rather than undermining it. If autonomy is to remain meaningful in an AI-mediated society, scientific models of agency must expand upstream to the decision level.

## 6 Empirical predictions and future research

The expanded three-level framework opens new avenues for empirical investigation. The following predictions delineate testable approaches for validating Decision-level Agency as a distinct construct and for examining its susceptibility to AI-mediated influence (Table 2). Taken together, these paradigms provide a foundation for establishing Decision-level Agency as an empirically tractable dimension of human volition.

**TABLE 2:** Testable predictions and experimental paradigms for Decision-, Action-, and Outcome-level Agency.

Prediction	Rationale	Testable paradigm
Prediction 1 (Dissociation): Disruption of prefrontal decision processes (e.g., via TMS/tDCS) will selectively reduce subjective Decision-level Agency while leaving Action- and outcome-level measures relatively intact.	Tests whether decision-level experience can be selectively altered without proportionally impairing movement execution or external outcome attribution.	Apply inhibitory TMS/tDCS over nodes supporting intention formation and commitment (e.g., pre-SMA, lateral PFC) during tasks requiring self-generated and self-paced intention commitment (e.g., covert commitment to one of two options) versus tasks where action selection is externally specified (e.g., cued button press) with comparable motor demands.
Prediction 2 (AI influence): AI-generated suggestions will selectively reduce Decision-level Agency ratings while leaving outcome-based measures unchanged.	Measures the specific vulnerability of the decision stage to external framing or nudging, addressing the framework’s ethical urgency.	Compare agency over self-generated choices versus choices adopted after being strongly suggested or framed by an “AI agent” in a covert decision task. The central hypothesis is that AI framing compromises the experience of origination and commitment, even if the subsequent action execution and its consequences remain controlled by the human agent.
Prediction 3 (Neural correlates): Neural signatures of intention commitment and stabilization (e.g., prefrontal activity, specific ERP components) will correlate with subjective Decision-level Agency.	Tests whether neural markers should distinguish high-agency decisions (self-generated, deliberate, committed) from low-agency decisions (externally influenced, forced-choice).	Record EEG/fMRI during self-paced, commitment-based decision tasks (where Decision-Level Agency is high) vs. forced-choice or externally framed decisions (where it is low). A key focus would be the temporal dynamics of prefrontal activity and/or ERP components indexing the stabilization of intention commitment.
Prediction 4 (Covert decisions): Tasks requiring covert decision commitment but no subsequent overt movement will still produce measurable agency signals.	Tests whether a purely mental action can anchor a sense of agency in the absence of motor execution and external outcomes.	To be developed. Participants make a self-paced internal decision between two alternatives (e.g., covertly commit to “option A” vs. “option B”) while withholding any motor response. Decision-level Agency can be assessed via explicit self-report (e.g., authorship/commitment ratings), and - where appropriate - via implicit temporal measures adapted to mental action–effect paradigms (e.g., belief-based action–effect designs).
Prediction 5 (Hierarchical interaction): Decision-level perturbations (e.g., abrupt intention revision) will propagate down the hierarchy, modulating the subsequent action-level prediction and the intensity of Outcome-level Agency.	Tests the hierarchical interaction predicted by the framework, whereby upstream instability affects downstream control and causal attribution.	Using computational modeling alongside behavioral tasks that require rapid, abrupt reversals of intention. Dissociable neural signals (e.g., in PFC vs. motor cortex) should track perturbations at the decision and action levels, with decision-level instability having a disproportionately larger impact on the final subjective experience of control.

## 7 Conclusion

The Sense of agency has traditionally been tethered to the external world, focusing on overt motor acts and their consequences. While this approach has generated valuable insights, it risks overlooking the internal, upstream processes through which autonomy is fundamentally exercised: the formation and stabilization of decisions and intentions. By formally recognizing deciding/intention formation as a mental action, the present work establishes Decision-level Agency (as defined here) as a distinct and theoretically useful dimension for organizing and extending existing accounts of SoA. The proposed three-level hierarchical framework (Decision → Action → Outcome) is offered as a conceptual clarification and measurement-oriented extension that makes explicit an often under-foregrounded locus of agency (intention formation and commitment). In doing so, it helps resolve conceptual ambiguities in existing models, aligns agency theory with contemporary neuroscientific evidence, and integrates with predictive processing accounts of hierarchical control.

This reconceptualization is therefore not only theoretically informative but also urgently relevant. As Generative AI systems increasingly influence human choices upstream before any physical action occurs, a science of agency that overlooks decision-level processes cannot adequately assess or safeguard autonomy. Advancing the science of agency requires shifting experimental attention upstream to the decision level. Doing so will not only refine theoretical models but also strengthen society’s capacity to preserve human autonomy in AI-mediated environments.
